# The Diffusion of Low-Energy Methyl Group on ITO Film Surface and Its Impact on Optical-Electrical Properties

**DOI:** 10.3390/ma11101991

**Published:** 2018-10-16

**Authors:** Shiping Zhao, Zhixuan Lv, Xuelin Guo, Chaoqian Liu, Hualin Wang, Weiwei Jiang, Shimin Liu, Nan Wang, Yunxian Cui, Wanyu Ding, Bing Han, Dongying Ju

**Affiliations:** 1 College of Materials Science and Engineering, Dalian Jiaotong University, Dalian 116028, China; zhaosp105@163.com (S.Z.); lzx_djtu@163.com (Z.L.); g1442944561@icloud.com (X.G.); cqliu@djtu.edu.cn (C.L.); whl@djtu.edu.cn (H.W.); jww@djtu.edu.cn (W.J.); lsm@djtu.edu.cn (S.L.); nwang@djtu.edu.cn (N.W.); dlcyx007@126.com (Y.C.); 2 Liaoning Province Key Laboratory of Metallurgical Equipment and Process Control, University of Science and Technology Liaoning, Anshan 114051, China; dyju@sit.ac.jp; 3 Advance Science Center, Saitama Institute of Technology, Fukaya 369-0293, Japan

**Keywords:** indium tin oxide, preferred orientation, optical and electrical property, diffusion, methyl group

## Abstract

Indium tin oxide (ITO) film is one of the ideal candidates for transparent conductive cathode in methylammonium lead halide perovskite solar cells. Thus, the diffusion of methyl group in ITO film is inevitable, which could deteriorate the optical-electrical property of ITO film. In this study, ITO films with and without (100) preferred orientation were bombarded by the low-energy methyl group beam. After the bombardment, the optical-electrical property of ITO film without (100) preferred orientation deteriorated. The bombardment of methyl group had little influence on the optical-electrical property of ITO film with (100) preferred orientation. Finally, combining the crystallographic texture and chemical bond structure analysis, the diffusion mechanism of low-energy methyl group on ITO lattice and grain boundary, as well as the relation between the optical-electrical property and the diffusion of the methyl group, were discussed systematically. With the above results, ITO film with (100) preferred orientation could be an ideal candidate for transparent conductive cathode in methylammonium lead halide perovskite solar cells.

## 1. Introduction

As one of the most spectacular solar cells, the methylammonium lead halide (MAPbH_3_) perovskite solar cell has attracted the attention of researchers from all over the world [1,2,3,4,5]. The typical MAPbH_3_ perovskite solar cell consists of the transparent conductive cathode, electron transfer layer, MAPbH_3_ perovskite layer, hole transport layer, and metal anode [6]. The electron transfer layer, MAPbH_3_ perovskite layer, and hole transport layer are focused on by most researchers [6,7,8,9,10,11,12]. To our knowledge, few studies have considered the transparent conductive cathode in MAPbH_3_ perovskite solar cells, such as indium tin oxide (ITO) film [13,14,15,16]. As the electrode in the MAPbH_3_ perovskite solar cell, ITO film suffers a serious attack from many kinds of groups/ions during the spin coating and annealing process of the MAPbH_3_ layer, such as methyl, amino, methylammonium, halide ion, and so on. The electron transfer layer could not completely prevent the attack on ITO film by the above groups/ions. Taking the methyl in precursor for instance, the methyl could exist as the methyl radical (${\mathrm{CH}}_{3}^{}$), methyl cation (${\mathrm{CH}}_{3}^{+}$), and methyl anion (${\mathrm{CH}}_{3}^{-}$), which could chemically diffuse on/into the ITO film surface. However, to the authors’ knowledge, there is little information available in literature about the diffusion of methyl on the ITO film surface [17]. In order to ensure the service life and stability of the MAPbH_3_ perovskite solar cell, except for the deterioration of the MAPbH_3_ layer, the deterioration of ITO cathode should also be considered. In our experiment, body-centered cubic (bcc) bixbyite ITO films with and without (100) preferred orientation were used as substrates. Then, the low-energy methyl group beam bombardment process was carried out onto the ITO film surface. Technically speaking, the low-energy methyl group beam bombardment was not equivalent to the annealing process that usually occurs during spin coating, but was equivalent to the artificial accelerated aging test. Through the low-energy methyl group beam bombardment process, the accumulation of methyl group diffused into the ITO film was simulated, which was caused by the spin coating MAPbH_3_ layer process and the service process of the MAPbH_3_ perovskite solar cell device. In general, the property of the ITO electrode was strongly influenced by the chemical composition and crystal structure, such as the optical-electrical properties and chemical stability. In the case of the MAPbH_3_ perovskite solar cell device, the commercial ITO electrode is mainly prepared using sputtering technology, which uses the ITO target with a particular chemical composition. Therefore, it was easy to keep the chemical composition of the commercial ITO film constant. Moreover, the crystal structure was the main factor that influenced the optical-electrical property and chemical stability of the ITO electrode in the MAPbH_3_ perovskite solar cell device. Therefore, this paper focuses on the diffusion process of the methyl group on the ITO film surface and its impact on the optical-electrical properties of ITO films, as well as the physicochemical process behind them. Moreover, special attention is paid to the mechanism according to which ITO film with (100) preferred orientation restrains the diffusion of the methyl group. 

## 2. Materials and Methods 

**Figure 1:**
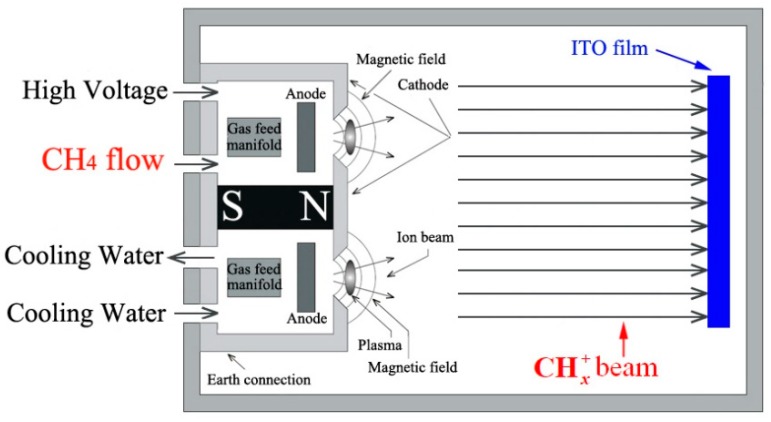
The schematic diagram of the processing chamber for ${\mathrm{CH}}_{x}^{+}$ beam bombardment (cross-section).

Firstly, four kinds of ITO films were prepared on the quartz substrate using the traditional direct current pulsed sputtering technique, with and without (100) preferred orientation. The thickness was fixed at 600 nm for all the ITO films. Detailed information about the ITO film deposition can be found in the Supplementary Materials. In the order without to with (100) preferred orientation, four kinds of ITO films were named as ITO film (i), (ii), (iii) and (iv), respectively. Then, the quartz substrates with ITO film were loaded into the cylindrical processing chamber. The basal diameter and height of the cylindrical processing chamber were 520 and 380 mm, respectively. The linear ion source was set at one side of the cylindrical processing chamber. The linear ion source was manufactured by Advanced Energy Industries, Inc., Washington D.C., USA. The quartz substrate with ITO film was loaded into the processing chamber, facing the ion source, as shown in Figure 1. The distance between the ion source and the substrate was fixed at 110 mm for all samples. After the base vacuum of the processing chamber was fixed at less than 9.9 × 10^−4^ Pa, high-purity methane (${\mathrm{CH}}_{4}^{}$, 99.999%) was introduced into the ion source. In this way, the ${\mathrm{CH}}_{3}^{+}$ beam was introduced into the processing chamber to bombard the surface of the ITO film. The gas flow ratio of ${\mathrm{CH}}_{4}^{}$ and working power of the ion source were fixed at 20 standard cubic centimeters per minute and 400 W, respectively. The anode voltage and current of the ion source were kept at 1.7 × 10^3^ V and 0.23 A for all experiments, respectively. By controlling the metal ultrahigh vacuum gat valve, the working pressure in the processing chamber was fixed at 0.4 Pa. The ITO film surface was bombarded without intentional heating to characterize its intrinsic physical and chemical properties. The bombardment time was fixed at 10 min for all samples. During the bombardment process, the temperature of the ITO film sample increased from room temperature (about 25 °C) to 50 °C, gradually. The detailed description was discussed in our previous publication [18,19]. Based on the plasma theory and the methane dehydrogenation process in plasma, methyl cation (${\mathrm{CH}}_{3}^{+}$), methylene cation (${\mathrm{CH}}_{2}^{+}$), and methine cation (${\mathrm{CH}}_{}^{+}$) were formed in the ion source [20,21,22,23]. Therefore, the methyl group beam contained the above three kinds of cation. In consideration of the dissociation energy, collision cross-section, and collision probability for the above three kinds of cation, ${\mathrm{CH}}_{3}^{+}$ was decidedly in the majority in the ion beam. To maintain a scientific and rigorous expression, the beam with the above three kinds of cation was expressed as the ${\mathrm{CH}}_{x}^{+}$ (1 ≤ *x* ≤ 3) beam in the manuscript.

The analytical techniques used in this study included X-ray diffraction (XRD, PANalytical Empyrean, Malvern Panalytical Ltd., Ettern Leur, The Netherlands), Hall Effect measurement, the use of an ultraviolet-visible (UV) spectrophotometer, (U-3310 UV, Hitachi, Ltd., Tokyo, Japan) and X-ray photoelectron spectroscopy (XPS, Quantum 2000, Physical Electronics, Inc., Chanhassen, MN, USA). The crystal structure and preferred orientation of the ITO film were analyzed using a PANalytical Empyrean XRD system, with Cu K_α1_ radiation (λ = 1.54056 Å), where the scanning mode, step size, and counting time were θ–2θ mode, 0.02°, and 0.5 s, respectively. The electrical property of the ITO film was measured using a Hall 8800 system at room temperature, with 0.68 T in magnetic flux density. The optical property of ITO film was determined using a U-3310 UV spectrophotometer, with 200–900 nm in scale and 2 nm in step. XPS spectra were performed ex-situ using a Quantum 2000 system with an Al K_α_ line source (*hv* = 1486.6 eV) at an incident angle of 45°. Before the measurement, the XPS system was calibrated using the standard Au and Cu samples. In consideration of the signal-to-noise ratio of the data, the area of XPS measurement was circular for all samples, with a diameter of 100 μm. Then, the high-resolution spectra were recorded with 29.35 and 0.125 eV in the pass energy and resolution, respectively. All the spectra were referenced to a C *1s* peak of 284.6 eV. In order to avoid the interference of surface contamination caused by an *ex-situ* XPS measurement, Ar ion (${\mathrm{Ar}}_{}^{+}$) bombardment was carried out using a differential pumping ion gun with 2 kV in accelerating voltage, 45° in incident angle, 4 min in bombardment time, which was a standard accessory function of the Quantum 2000 XPS system. Based on the semi-empirical data, the sputtering ratio of the ${\mathrm{Ar}}_{}^{+}$ differential pumping ion gun with the above sputtering parameters was about 5–10 nm/min. After 4 min of ${\mathrm{Ar}}_{}^{+}$ sputtering, the depth represented by the XPS spectra was about 20–40 nm under the surface. Based on our previous results, the surface morphology of all the ITO films was uniform and the root-mean-square roughness of all the films was lower than 3 nm. Compared with the sputtering depth of 20–40 nm, the root-mean-square roughness of 3 nm could not influence the sputtering ratio during the XPS measurement. Also, it takes similar amount of time to remove the surface layer of all the ITO films during the XPS measurement. Detailed information about the surface morphology of ITO film can be found in the Supplementary Materials.

## 3. Results and Discussion

### 3.1. Crystallographic Texture Analysis

**Figure 2:**
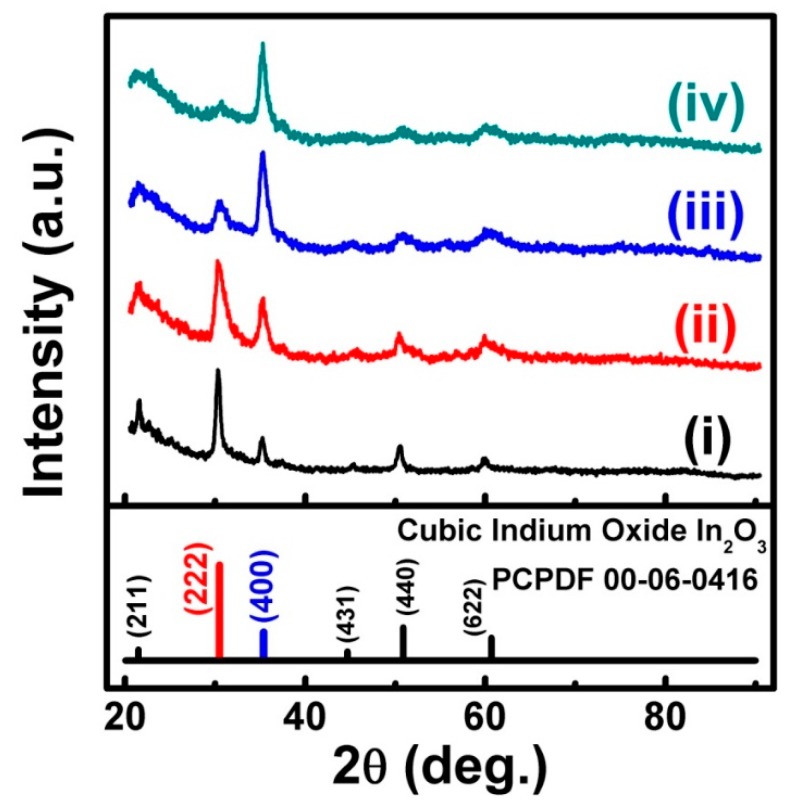
XRD patterns of ITO films before ${\mathrm{CH}}_{x}^{+}$ beam bombardment.

In the experiment, four kinds of ITO films were selected, which were named ITO film (i), (ii), (iii), and (iv), respectively. To confirm the crystallographic texture of ITO film (i), (ii), (iii), and (iv), an XRD measurement with a θ–2θ scanning mode was carried out on all the ITO films before and after ${\mathrm{CH}}_{x}^{+}$ beam bombardment. Figure 2 shows the normalized XRD patterns of all the ITO films before ${\mathrm{CH}}_{x}^{+}$ beam bombardment. From Figure 2, it is clear that the XRD patterns of ITO films (i) to (iv) display diffraction peaks attributed to bcc bixbyite In_2_O_3_ (PCPDFWIN card number: 00-06-0416 (Version 2.1, Copyright 2000)) [24,25]. Moreover, it should be noted that the XRD pattern of ITO film (i) is similar to that of bcc bixbyite In_2_O_3_ standard polycrystalline powder. For ITO films (ii), (iii), and (iv), the XRD patterns display an increasingly obvious (400) (the same as (100)) preferred orientation, because the relative intensity of the (400) peaks became increasingly strong compared to those of the (222) and (440) peaks. Especially for ITO film (iv), almost only a (400) diffraction peak could be found, which means ITO film (iv) with a (100) preferred orientation. The XRD patterns of the ITO films after ${\mathrm{CH}}_{x}^{+}$ beam bombardment were similar to those of the ITO film before ${\mathrm{CH}}_{x}^{+}$ beam bombardment. It is easy to understand that the bombardment temperature was only about 50 °C, which could not influence the crystal structure of the ITO film, or the XRD patterns.

### 3.2. Optical-Electrical Property Analysis

**Figure 3:**
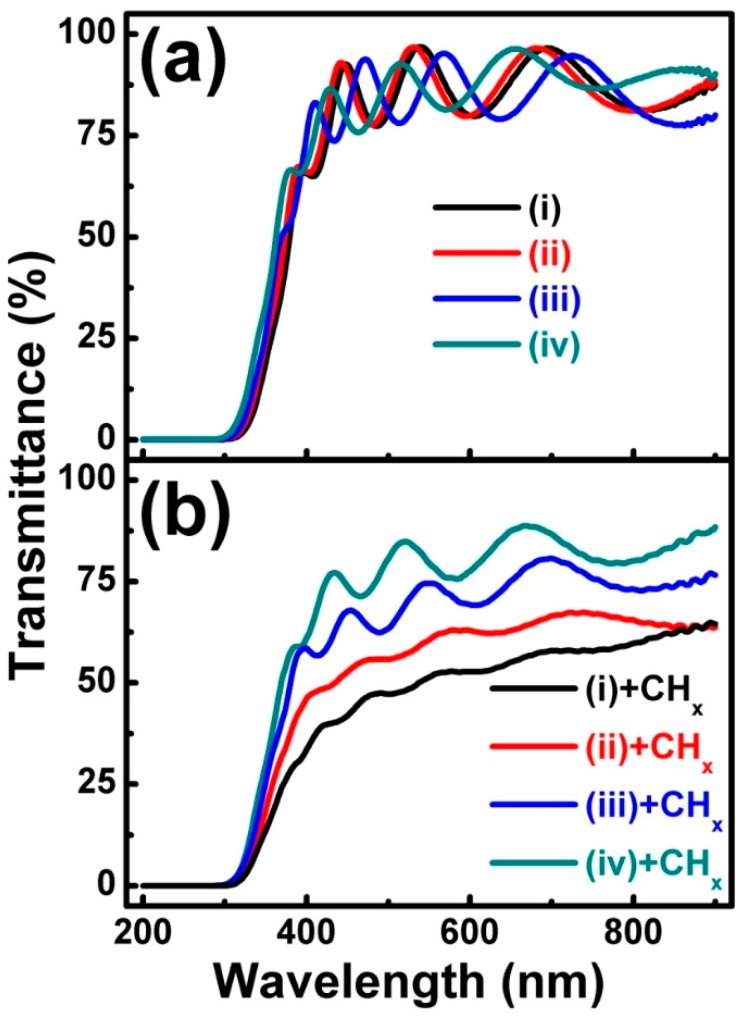
The transmission spectra of ITO films before (**a**) and after (**b**) ${\mathrm{CH}}_{x}^{+}$ beam bombardment.

In order to understand the relation between the diffusion of the low-energy ${\mathrm{CH}}_{x}^{+}$ group on the ITO film surface and its impact on optical-electrical properties, a transmission spectra measurement was carried out on the ITO films before and after ${\mathrm{CH}}_{x}^{+}$ beam bombardment, as shown in Figure 3. From Figure 3a, it is clear that for ITO films (i) to (iv), the transmission spectra are similar and the average transmittance in the visible range (400-800 nm) of all the ITO films was kept at (86.36 ± 0.35)%. Figure 3b shows the transmission spectra of the ITO films bombarded by the ${\mathrm{CH}}_{x}^{+}$ beam, which are different from those in Figure 3a. Firstly, after ${\mathrm{CH}}_{x}^{+}$ beam bombardment, the transmittance of all the ITO films evidently decreased. The average transmittance in the visible range (400–800 nm) of ITO films (i) to (iv) in Figure 3b decreased to 51.57%, 60.58%, 71.16%, and 79.90%, respectively. Secondly, after ${\mathrm{CH}}_{x}^{+}$ beam bombardment, a red-shift of the optical absorption edge could be clearly observed. More importantly, for ITO films (i) to (iv), the decrease in transmittance becomes increasingly slight. Combining the XRD and transmission spectra results, it seems that with the crystallographic texture of the ITO film from no preferred orientation to a (100) preferred orientation, the decrease in transmittance becomes increasingly slight, which is caused by ${\mathrm{CH}}_{x}^{+}$ beam bombardment.

**Figure 4:**
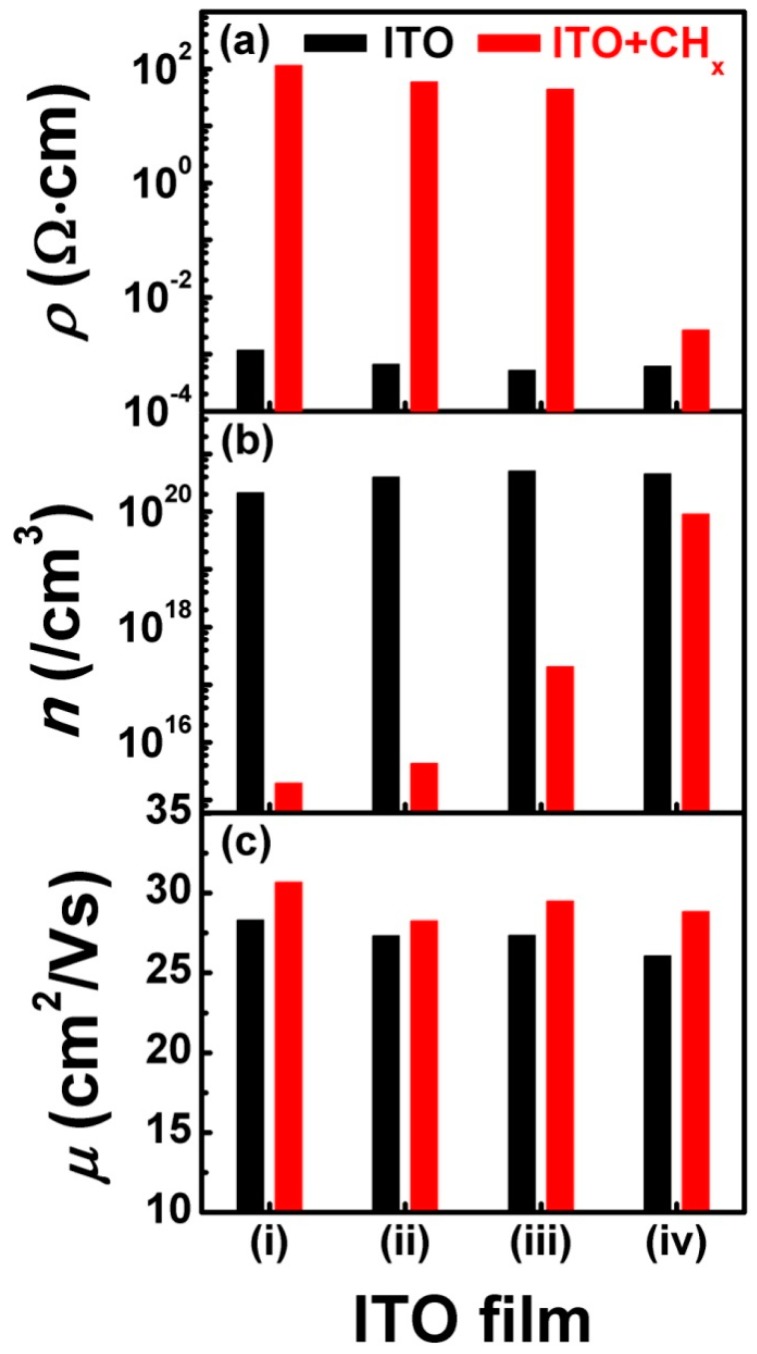
The electronic property of ITO films before and after ${\mathrm{CH}}_{x}^{+}$ beam bombardment. (**a**) for resistivity; (**b**) for carrier concentration and (**c**) for carrier mobility.

Except for the optical property, the electrical property of the ITO film was also investigated using the Hall Effect measurement, as shown in Figure 4. The results show that the resistivity of the original ITO films was kept at ~10^−4^ Ω·cm. After ${\mathrm{CH}}_{x}^{+}$ beam bombardment, the resistivity of the ITO films—except for that of ITO film (iv), which slightly increased to 2.7 × 10^−3^ Ω·cm—increased by 6 orders of magnitude to ~10^2^ Ω·cm. Meanwhile, the carrier concentration of the original ITO films was kept at ~10^20^/cm^3^. After ${\mathrm{CH}}_{x}^{+}$ beam bombardment, the carrier concentration of the ITO films—except for that of ITO film (iv), which was kept at 10^20^/cm^3^—decreased to ~10^15–17^/cm^3^. The carrier mobility of the ITO films was kept at 23.71 ± 1.09 cm^2^/Vs. After ${\mathrm{CH}}_{x}^{+}$ beam bombardment, the carrier mobility slightly increased to 26.30 ± 1.38 cm^2^/Vs. Similarly, combining the XRD and Hall results, it seems that with the crystallographic texture of the ITO film moving from no preferred orientation to a (100) preferred orientation, the deterioration of resistivity and carrier concentration became increasingly slight, which was caused by ${\mathrm{CH}}_{x}^{+}$ beam bombardment.

Moreover, it seems that the different deterioration of optical-electrical properties occurred on the ITO film with and without a (100) preferred orientation, which was caused by ${\mathrm{CH}}_{x}^{+}$ beam bombardment. So, two questions are raised. One is why low-energy ${\mathrm{CH}}_{x}^{+}$ beam bombardment could deteriorate the optical-electrical properties of the ITO film. The other is why a (100) preferred orientation could effectively restrain the deterioration of ITO film optical-electrical properties caused by ${\mathrm{CH}}_{x}^{+}$ beam bombardment. With an understanding of these two questions, ITO film with an ideal crystallographic texture could be securely used as the cathode in MAPbH_3_ perovskite solar cells. 

### 3.3. XPS Analysis

In general, the optical-electrical properties of ITO film are strongly influenced by chemical composition and chemical bond structure [26]. In this study, all the ITO films were prepared using sputtering technology, which used an ITO ceramic target and pure Ar sputtering. Detailed information about ITO film deposition can be found in the Supplementary Materials. Based on our previous report, the chemical composition and chemical bond structure of ITO films deposited by an ITO ceramic target and pure Ar sputtering were kept at constant, irrespective of what the crystallographic texture was [27]. Moreover, the optical-electrical properties of all the original ITO films were kept at constant, which corresponded well with their constant chemical composition and chemical bond structure. Moreover, the discussion should be focused on the chemical composition and chemical bond structure of ITO films bombarded by the ${\mathrm{CH}}_{x}^{+}$ beam.

**Figure 5:**
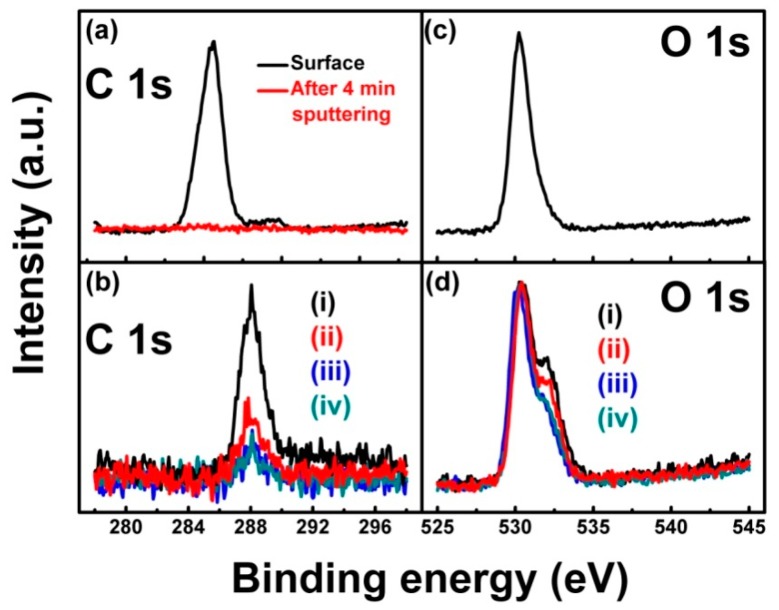
High-resolution XPS spectrum. (**a**) C 1*s* for ITO film (i) before ${\mathrm{CH}}_{x}^{+}$ beam bombardment; (**b**) C 1*s* for ITO films (i) to (iv) bombarded by ${\mathrm{CH}}_{x}^{+}$ beam, after 4 min of ${\mathrm{Ar}}_{}^{+}$ cleaning; (**c**) O 1*s* for ITO film (i) before ${\mathrm{CH}}_{x}^{+}$ beam bombardment, after 4 min of ${\mathrm{Ar}}_{}^{+}$ cleaning and (**d**) O 1*s* for ITO films (i) to (iv) bombarded by ${\mathrm{CH}}_{x}^{+}$ beam, after 4 min of ${\mathrm{Ar}}_{}^{+}$ cleaning.

To understand the above two questions about the reaction between ITO film and ${\mathrm{CH}}_{x}^{+}$ beam bombardment, a high-resolution XPS measurement was carried out. Figure 5 displays the high-resolution XPS spectra of C 1*s* and O 1*s* for ITO film before and after ${\mathrm{CH}}_{x}^{+}$ beam bombardment. At a position of 284.6 eV, a strong C 1*s* peak appears on the surface of ITO film (i), which is attributed to the organic contaminant caused by the *ex-situ* XPS measurement, as shown in Figure 5a [28,29]. Of course, after 4 min of ${\mathrm{Ar}}_{}^{+}$ cleaning in the XPS measurement chamber, the C 1*s* peak disappears completely. Therefore, the organic contaminant is only adsorbed onto the surface of the ITO film. For ITO films (i) to (iv) bombarded by a ${\mathrm{CH}}_{x}^{+}$ beam, after 4 min of ${\mathrm{Ar}}_{}^{+}$ cleaning in the XPS measurement chamber, a new C 1*s* peak appears at 288.2 eV, which is attributed to the O–C–O bonds, as shown in Figure 5b [29,30]. Comparing Figure 5a with Figure 5b, it can be seen that after ${\mathrm{CH}}_{x}^{+}$ beam bombardment, the ${\mathrm{CH}}_{x}^{+}$ groups diffused onto the ITO film. Meanwhile, for ITO film (i), after 4 min of ${\mathrm{Ar}}_{}^{+}$ cleaning in the XPS measurement chamber, at a position of 530.1 eV, only one O 1*s* peak appeared on the film surface, which is attributed to the In/Sn–O bond, as shown in Figure 5c. Unsurprisingly, after ${\mathrm{CH}}_{x}^{+}$ beam bombardment, another weak shoulder O 1*s* peak appears at 532.2 eV, which is attributed to the In/Sn–O–C and O–C–O bonds, as shown in Figure 5d [28,29]. More importantly, based on Figure 5c,d, it should be noted that in the order of ITO films (i) to (iv) bombarded by a ${\mathrm{CH}}_{x}^{+}$ beam, the intensity of the C 1*s* peak and the shoulder peak of O 1*s* both decreased, monotonously. 

**Figure 6:**
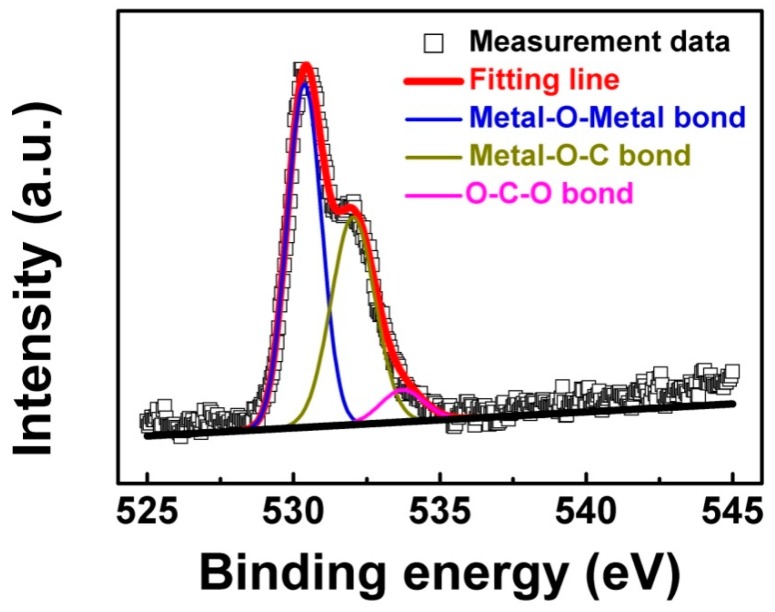
Gaussian peak fitting procedure applied to O 1*s* high-resolution XPS spectrum of ITO film (i) bombarded by a ${\mathrm{CH}}_{x}^{+}$ beam, after 4 min of ${\mathrm{Ar}}_{}^{+}$ cleaning.

**Figure 7:**
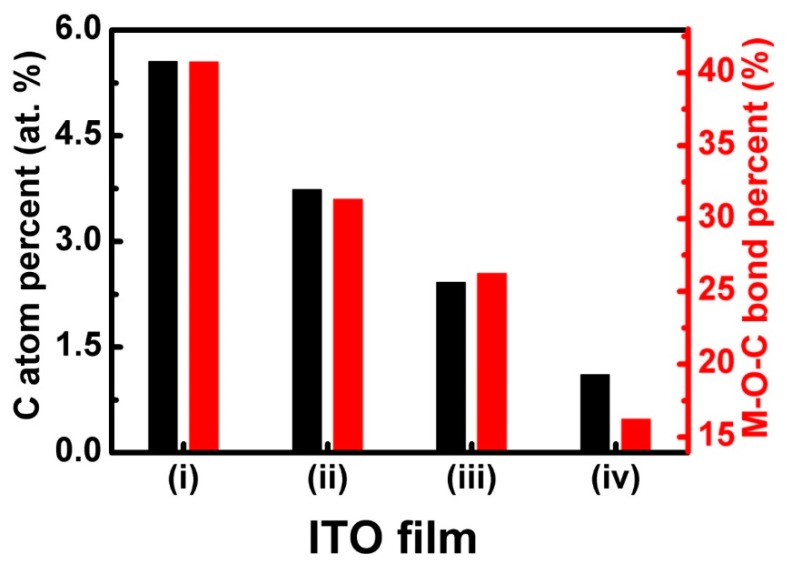
The atomic percentage of C and percentage M–O–C bond from all O bonds.

To investigate the detailed information about O 1*s* high-resolution XPS spectra, the Gaussian peak fitting procedure was carried out for the O 1*s* high-resolution XPS spectra of the ITO film bombarded by a ${\mathrm{CH}}_{x}^{+}$ beam. Figure 6 shows the Gaussian peak fitting result for ITO film (i) bombarded by a ${\mathrm{CH}}_{x}^{+}$ beam, after 4 min of ${\mathrm{Ar}}_{}^{+}$ cleaning. From Figure 6, it can been seen that the Gaussian peak fitting result shows three peaks located at 530.1, 532.0, and 533.7 eV, which are attributed to In/Sn–O–In/Sn (Metal–O–Metal, M–O–M), In/Sn–O–C (Metal–O–C, M–O–C), and O–C–O bonds, respectively [28,29]. The percentages of each kind of O bond from all O bonds could be calculated using the semi-quantitative Equation (1) [28,29]:

(1) $$A_{x}=\frac{I_{x}}{\sum I_{i}},$$

where A*_x_* and I*_x_* are the percentages of certain bond structures from an element and the integrated area of certain bond structure of an element XPS characteristic peak, respectively. The calculated results show that the sum percentage of O–C–O bonds stayed at (7.3 ± 1.1)% for all ITO films bombarded by a ${\mathrm{CH}}_{x}^{+}$ beam. Meanwhile, the percentage of the M–O–C bond decreased lineally from 40.8 to 16.3% for ITO films (i) to (iv) bombarded by a ${\mathrm{CH}}_{x}^{+}$ beam, as shown in Figure 7. Meanwhile, to quantitatively analyze the XPS results, the atomic percent of C was calculated using the semi-quantitative Equation (2) [28,29]:

(2) $$A_{C}=\frac{\frac{I_{C}}{S_{C}}}{\sum \frac{I_{i}}{S_{i}}},$$

where A_C_, I_C_, S_C__,_ I*_i_*, and S*_i_* are the atomic percentages of C, the integrated area of C 1*s* peak, the sensitivity factors of C, the integrated area of the characteristic peak of a certain element, and the sensitivity factors of a certain element, respectively. The calculated results are shown in Figure 7. It can be found that the atomic percentage of C decreased lineally from 5.6 to 1.1 at % for ITO films (i) to (iv) bombarded by a ${\mathrm{CH}}_{x}^{+}$ beam. 

Based on the XPS results in Figure 5, Figure 6 and Figure 7, the question about the serious deterioration of the optical-electrical properties of ITO film caused by the bombardment of a low-energy ${\mathrm{CH}}_{x}^{+}$ beam could be discussed. Firstly, during the low-energy ${\mathrm{CH}}_{x}^{+}$ beam bombardment process, the ${\mathrm{CH}}_{x}^{+}$ group could chemically diffuse on the ITO film surface, which contains a C– dangling bond. Secondly, in this study, the ${\mathrm{CH}}_{x}^{+}$ beam directionally bombarded the ITO film surface, which was the normal incidence, as shown in Figure 1. Moreover, the ${\mathrm{CH}}_{x}^{+}$ group could not only chemically diffuse on the ITO film surface, but could also chemically diffuse into the ITO film surface along the normal direction. Thirdly, based on the XRD result in Figure 2, the ITO films used in experiment had a polycrystalline structure, which means the ITO film mainly consists of ITO grains. In the diffusion system of (${\mathrm{CH}}_{x}^{+}$ group) → (ITO lattice and grain boundary), the ${\mathrm{CH}}_{x}^{+}$ group preferentially diffuses at grain boundary if the ${\mathrm{CH}}_{x}^{+}$ group was of a small amount. In the case of an excess ${\mathrm{CH}}_{x}^{+}$ group, diffusion within the ITO grain has to be considered. Moreover, the ${\mathrm{CH}}_{x}^{+}$ group could diffuse into the ITO lattice, as well as the grain boundary between the grains. More importantly, the ${\mathrm{CH}}_{x}^{+}$ group is not chemically stable in the ITO lattice, because of the chemical activity of the C– dangling bond. Finally, it is necessary for the C– dangling bond in the ${\mathrm{CH}}_{x}^{+}$ group to chemically react with the element in the ITO lattice and grain boundary. At room temperature, it is very difficult for the C– dangling bond in ${\mathrm{CH}}_{x}^{+}$ group to chemically react with In/Sn and to form metal carbides [30,31]. Compared with In/Sn, it is more likely for the C– dangling bond in the ${\mathrm{CH}}_{x}^{+}$ group to chemically react with O in the ITO lattice and grain boundary. Therefore, a great deal of M–O–C bonds appear in high-resolution XPS spectra of O 1s, as shown in Figure 5, Figure 6 and Figure 7. During 10 min of ${\mathrm{CH}}_{x}^{+}$ beam bombardment, there is little probability that the C– dangling bond in the ${\mathrm{CH}}_{x}^{+}$ group will chemically react with O to form the O–C–O bond in the ITO lattice and grain boundary, which results in the constant percentage of the O–C–O bond as (7.3 ± 1.1)%.

Based on the above discussion, the change in the optical-electrical properties of the ITO film in Figure 3 and Figure 4 can also be partially understood. After ${\mathrm{CH}}_{x}^{+}$ beam bombardment, a larger number of ${\mathrm{CH}}_{x}^{+}$ groups chemically react with O in the ITO lattice and grain boundary, and M–O–C bonds form in the ITO lattice and grain boundary. It is well-known that Sn^4+^ and O vacancy are the key factors of the ITO film conduction mechanism [26,27]. The formation of M–O–C bonds in the ITO lattice and grain boundary inevitably decreases the O vacancy, which results in the deterioration of *n*. The kinetic energy of ${\mathrm{CH}}_{x}^{+}$ groups in our experiment was not high enough to change the distribution of the grain boundary in the ITO film, which scatters the electrons during the Hall measurement. Moreover, little change occurs on the carrier mobility of the ITO film before and after the ${\mathrm{CH}}_{x}^{+}$ beam bombardment. As a result, the optical-electrical property displays deterioration, as shown in Figure 3 and Figure 4. However, the above discussion cannot solve the second question, regarding why the (100) preferred orientation could effectively restrain the deterioration of ITO film optical-electrical property caused by ${\mathrm{CH}}_{x}^{+}$ beam bombardment.

### 3.4. ${\mathrm{CH}}_{x}^{+}$ Diffusion Analysis

**Figure 8:**
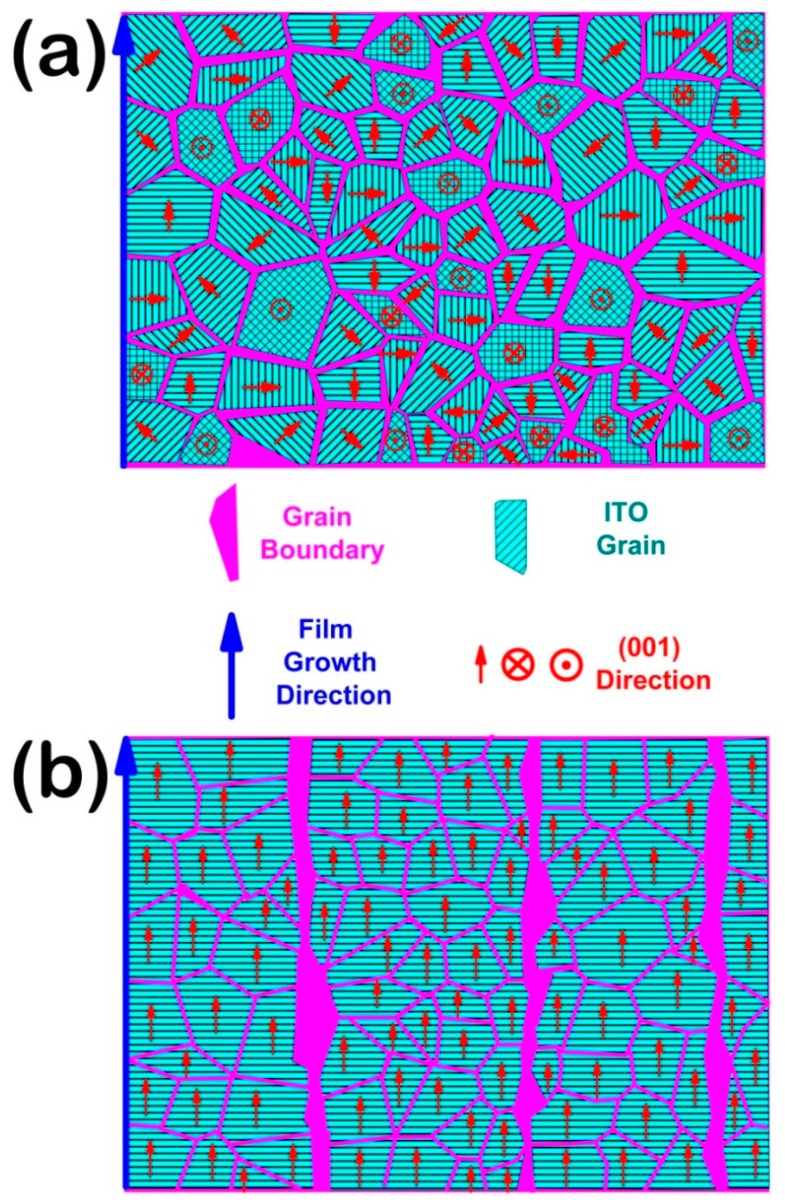
The cross-section diagrammatic sketch of the ITO film. (**a**) For polycrystalline without a preferred orientation; (**b**) for polycrystalline with a (100) preferred orientation.

Based on the film growth theory, whether with or without a (100) preferred orientation, the polycrystalline ITO film contains many grain boundaries [32,33,34,35], as shown in Figure 8a,b. Moreover, the ${\mathrm{CH}}_{x}^{+}$ group diffuses on the ITO grain boundary, as well as into the ITO grain. Combining the XRD and XPS results, the diffusion process of ${\mathrm{CH}}_{x}^{+}$ groups in the ITO film, as well as the restraint mechanism of the (100) preferred orientation for ${\mathrm{CH}}_{x}^{+}$ diffusion, could be discussed. 

Firstly, the diffusion of the ${\mathrm{CH}}_{x}^{+}$ group in the ITO grain boundary is discussed. The grain boundary consists of an amorphous structure, which contains many O– dangling bonds. In general, such O– dangling bonds at grain boundary could react with the ${\mathrm{CH}}_{x}^{+}$ diffused into the ITO film, which could decrease the vacancy and absorb the free electrons in the film. Then, the decrease in vacancy and absorption of free electrons at grain boundary could decrease the carrier concentration of the ITO film bombarded by a ${\mathrm{CH}}_{x}^{+}$ beam, which increases the resistivity of the film. The carrier mobility is mainly influenced by the electron scattering at grain boundary. Whether with or without a (100) preferred orientation, the polycrystalline ITO film contains a similar grain boundary density. Moreover, the grain boundary density is not influenced by the ${\mathrm{CH}}_{x}^{+}$ bombardment, which results in little change to the carrier mobility before and after ${\mathrm{CH}}_{x}^{+}$ bombardment. 

**Figure 9:**
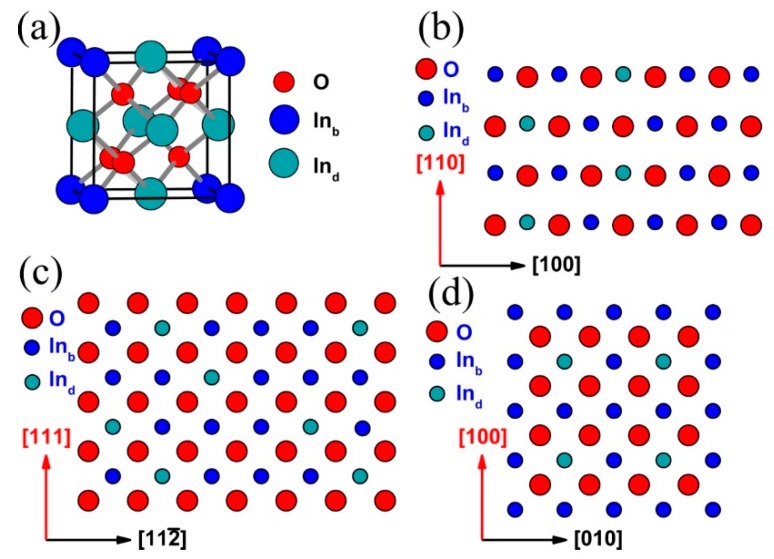
Schematic illustration of an undistorted bcc bixbyite In_2_O_3_ unit cell. (**a**) Undistorted primitive unit cell; (**b**) Schematic illustration of side view unrelaxed clean (110) surface; (**c**) Schematic illustration of side view unrelaxed clean (111) surface; (**d**) Schematic illustration of side view unrelaxed clean (100) surface.

Secondly, the diffusion of the ${\mathrm{CH}}_{x}^{+}$ group in the ITO grain is discussed. To understand the restraint mechanism of the (100) preferred orientation for ${\mathrm{CH}}_{x}^{+}$ diffusion, the property of the ITO lattice, as well as the reaction of the ${\mathrm{CH}}_{x}^{+}$ groups on the ITO lattice, should also be considered. The lattice of the ITO is the same as that of In_2_O_3_. For convenience, only the In_2_O_3_ lattice is discussed here. One In_2_O_3_ conventional unit cell consists of 32 In atoms and 48 O atoms [36,37]. One In_2_O_3_ conventional unit cell is more like a distorted version of a hypothetical parent crystal, in which 32 In atoms arrange as a face-centered cubic lattice and 48 O atoms occupy all the tetrahedral interstitial sites. For 32 In atoms in one In_2_O_3_ conventional unit cell, these are commonly referred to as In_b_ (8 atoms per conventional unit cell) and In_d_ (24 atoms per conventional unit cell). In_b_ sites are axially symmetrically coordinated, and In_d_ sites present high asymmetry in their coordination. Figure 9a shows the simplest schematic illustration of the undistorted bcc bixbyite In_2_O_3_ primitive unit cell. Based on Figure 9a, the side views of the three main lattices with a low Miller index are built, which are (111), (110), and (100), respectively. Figure 9b–d show the schematic illustration of the side view unrelaxed clean (111), (110), and (100) surface. 

In one conventional In_2_O_3_ unit cell, 75% of the In atoms occupy the positions of In_d_. Therefore, in the ITO lattice, Sn^4+^ is more likely to occupy the position of In_d_. From Figure 9b–d, it can be seen that the outer layers of the (111), (110) and (100) lattice are terminated by O, O/In(Sn), and In(Sn), respectively. From the aspect of termination at the outer layer, the ${\mathrm{CH}}_{x}^{+}$ group prefers to diffuse at the (111) and (110) lattice of the ITO grain, because of the abundance of O at these lattices. From the aspect of termination at the outer layer, it is difficult for the ${\mathrm{CH}}_{x}^{+}$ group to diffuse at athe (100) lattice of the ITO grain, because of the absence of O at the (100) lattice. Moreover, the calculated surface energy (*γ*, J/m^2^) of the prominent facets with low Miller index In_2_O_3_ unit cells shows that *γ*_{100}/{010}/{001}_, *γ*_{110}/{101}/{011}_, and *γ*_{111}_ are 1.76, 1.07 and 0.89 J/m^2^, respectively [38]. From the aspect of surface energy, the ${\mathrm{CH}}_{x}^{+}$ group prefers to diffuse at the (111) and (110) lattice, rather than the (100) lattice.

Except for the above discussion, the ${\mathrm{CH}}_{x}^{+}$ beam bombardment process should also be considered. In our experiment, a ${\mathrm{CH}}_{x}^{+}$ beam was introduced into the processing chamber through a collimating slit. Combining the small distance (110 mm) between the outlet of the ${\mathrm{CH}}_{x}^{+}$ beam and the ITO film surface, the incident angle of the ${\mathrm{CH}}_{x}^{+}$ beam to the ITO film surface was limited to ± 10^°^ from the normal direction of the film surface. Therefore, the ${\mathrm{CH}}_{x}^{+}$ beam bombardment technique used in the manuscript belongs to the plasma source ion beam bombardment method, which is different from plasma immersion ion bombardment. The most important advantage of the former is that it overcomes the heterogeneity of the bombardment, which is caused by the larger range of the ion incident angle. Technically, the homogeneous incident angle of the ${\mathrm{CH}}_{x}^{+}$ beam ensures the homogeneous diffusion of the ${\mathrm{CH}}_{x}^{+}$ group on the ITO surface. Moreover, the homogeneous incident angle of the ${\mathrm{CH}}_{x}^{+}$ beam could avoid the shadow effect caused by the surface roughness. From the aspect of plasma source ion beam bombardment, for ITO film without a preferred orientation, during the ${\mathrm{CH}}_{x}^{+}$ group diffusion process, the probability is the same for the ${\mathrm{CH}}_{x}^{+}$ group to diffuse at any lattices with a low Miller index. In consideration of the termination at the outer layer and the surface energy of the above lattices with a low Miller index, the ${\mathrm{CH}}_{x}^{+}$ group could chemically diffuse at the (111) and (110) lattices, which results in a larger number of M–O–C bonds formed at these lattices, as demonstrated by the XPS results shown in Figure 5, Figure 6 and Figure 7. From the aspect of plasma source ion beam bombardment, for an ITO film with a (100) preferred orientation, during the ${\mathrm{CH}}_{x}^{+}$ group diffusion process, the probability is much higher for the ${\mathrm{CH}}_{x}^{+}$ group to diffuse at (100) lattices than at any other lattices with a low Miller index. In consideration of the termination at the outer layer and the surface energy of (100) lattices, the ${\mathrm{CH}}_{x}^{+}$ group undergoes little change to chemically diffuse at the (100) lattice, which results in few M–O–C bonds formed at the (100) lattices, as demonstrated by the XPS results shown in Figure 5, Figure 6 and Figure 7. 

Based on the above analysis, the diffusion of the ${\mathrm{CH}}_{x}^{+}$ group in the grain boundary is similar for an ITO film with and without a (100) preferred orientation, which causes a similar deterioration in its optical-electrical properties. From the aspect of termination at the outer layer, surface energy, and plasma source ion beam bombardment, the ${\mathrm{CH}}_{x}^{+}$ group prefers to chemically react at (111) and (110) lattices rather than at (100) lattice, which causes a different deterioration of the optical-electrical properties of ITO film with and without a (100) preferred orientation. Combining the above two reasons, an ITO film with a (100) preferred orientation could effectively restrain the diffusion of the ${\mathrm{CH}}_{x}^{+}$ group.

## 4. Conclusions

In summary, traditional indium tin oxide (ITO) film was bombarded by a low-energy methyl cation (${\mathrm{CH}}_{x}^{+}$) beam. Special attention was paid to the relation between the optical-electrical properties of the ITO film and the (100) preferred orientation of the ITO film, which was bombarded by a ${\mathrm{CH}}_{x}^{+}$ beam. After ${\mathrm{CH}}_{x}^{+}$ beam bombardment, the optical-electrical properties of ITO films without a (100) preferred orientation deteriorated seriously. After ${\mathrm{CH}}_{x}^{+}$ beam bombardment, almost no deterioration of optical-electrical properties occurred on ITO film with a (100) preferred orientation. The XPS results reveal that after ${\mathrm{CH}}_{x}^{+}$ beam bombardment, a large number of metal–O–C bonds were formed in ITO films without a (100) preferred orientation, which resulted in the deterioration of their optical-electrical properties. Unsurprisingly, almost no metal–O–C bonds were formed in the ITO film with a (100) preferred orientation, which results in little change to its optical-electrical properties. Combining the XRD and XPS results, as well as the property of the ITO lattice, it can be concluded that the diffusion of the ${\mathrm{CH}}_{x}^{+}$ group at the grain boundary is similar for an ITO film with and without a (100) preferred orientation. In the case of ITO crystal grain, the ${\mathrm{CH}}_{x}^{+}$ group prefers to chemically diffuse at (111) and (110) lattices, rather than at (100) lattice. For the above reasons, an ITO film with a (100) preferred orientation could effectively restrain the diffusion of the ${\mathrm{CH}}_{x}^{+}$ group.

Finally, to apply ITO film to a MAPbH_3_ perovskite solar cell, it is of interest to avoid the deterioration in optical-electrical properties caused by the diffusion of ${\mathrm{CH}}_{x}^{+}$ during the preparation process of the MAPbH_3_ perovskite layer. This study reveals the restraint mechanism of a (100) preferred orientation for ${\mathrm{CH}}_{x}^{+}$ diffusion, as well as the physicochemical mechanisms occurring behind the diffusion process. Based on the results reported, an ITO film with a (100) preferred orientation could be an ideal choice for a transparent conductive cathode in MAPbH_3_ perovskite solar cells.

## Supplementary Materials

The following are available online at http://www.mdpi.com/1996-1944/11/10/1991/s1, Figure S1: The cross-section TEM image of ITO film (iv) on Si (100) substrate; Figure S2: AFM images for ITO film (iv). (a) and (b) are the surface morphology with planar and 3D AFM images respectively, which the scanning size is 1 × 1 μm^2^. (c) and (d) are the surface morphology with planar and 3D AFM images respectively, which the scanning size is 16 × 16 μm^2^; Table S1: The detailed parameters for ITO film deposition.

## References

[1] Kojima A., Teshima K., Shirai Y., Miyasaka T. (2009). Organometal Halide Perovskites as Visible-Light Sensitizers for Photovoltaic Cells. J. Am. Chem. Soc..

[2] Burschka J., Pellet N., Moon S.J., Humphry-Baker R., Gao P., Nazeeruddin M.K., Grätzel M. (2013). Sequential deposition as a route to high-performance perovskite-sensitized solar cells. Nature.

[3] Nie W., Tsai H., Asadpour R., Blancon J.C., Neukirch A.J., Gupta G., Crochet J.J., Chhowalla M., Tretiak S., Alam M.A. (2015). High-efficiency solution-processed perovskite solar cells with millimeter-scale grains. Science.

[4] Dymshits A., Iagher L., Etgar L. (2016). Parameters Influencing the Growth of ZnO Nanowires as Efficient Low Temperature Flexible Perovskite-Based Solar Cells. Materials.

[5] Liu M., Johnston M.B., Snaith H.J. (2013). Efficient planar heterojunction perovskite solar cells by vapour deposition. Nature.

[6] Green M.A., Ho-Baillie A., Snaith H.J. (2014). The emergence of perovskite solar cells. Nat. Photonics.

[7] Lee M.M., Teuscher J., Miyasaka T., Murakami T.N., Snaith H.J. (2012). Efficient hybrid solar cells based on meso-superstructured organometal halide perovskites. Science.

[8] Xing G.C., Mathews N., Sun S.Y., Lim S.S., Lam Y.M., Gratzel M., Mhaisalkar S., Sum T.C. (2013). Long-range balanced electron- and hole-transport lengths in organic-inorganic CH_3_NH_3_PBI_3_. Science.

[9] Yang W.S., Noh J.H., Jeon N.J., Kim Y.C., Ryu S., Seo J., Seok S.I. (2015). High-performance photovoltaic perovskite layers fabricated through intramolecular exchange. Science.

[10] Jeon N.J., Noh J.H., Kim Y.C., Yang W.S., Ryu S., Seok S.I. (2014). Solvent engineering for high-performance inorganic–organic hybrid perovskite solar cells. Nat. Mater..

[11] Jeon N.J., Noh J.H., Yang W.S., Kim Y.C., Ryu S., Seo J., Seok S.I. (2015). Compositional engineering of perovskite materials for high-performance solar cells. Nature.

[12] Heo J.H., Im S.H., Noh J.H., Mandal T.N., Lim C.S., Chang J.A., Lee Y.H., Kim H., Sarkar A., Nazeeruddin M.K. (2013). Efficient inorganic–organic hybrid heterojunction solar cells containing perovskite compound and polymeric hole conductors. Nat. Photonics.

[13] Liu D., Kelly T.L. (2014). Perovskite solar cells with a planar heterojunction structure prepared using room-temperature solution processing techniques. Nat. Photonics.

[14] Zhou H., Chen Q., Li G., Luo S., Song T., Duan H.S., Hong Z., You J., Liu Y., Yang Y. (2014). Interface engineering of highly efficient perovskite solar cells. Science.

[15] Gwamuri J., Marikkannan M., Mayandi J., Bowen P.K., Pearce J.M. (2016). Influence of oxygen concentration on the performance of ultra-thin RF magnetron sputter deposited indium tin oxide films as a top electrode for photovoltaic devices. Materials.

[16] Dunkel C., von Graberg T., Smarsly B.M., Oekermann T., Wark M. (2014). Limits of ZnO electrodeposition in mesoporous tin doped indium oxide films in view of application in dye-sensitized solar cells. Materials.

[17] Lin S., Xie D. (2012). Initial decomposition of methanol and water on In_2_O_3_ (110): A periodic DFT study. Chin. J. Chem..

[18] Ding W., Ju D., Chai W. (2010). The effect of working pressure on the chemical bond structure and hydrophobic property of PET surface treated by N ion beams bombardment. Appl. Surf. Sci..

[19] Ding W., Li L., Zhang L., Ju D., Peng S., Chai W. (2013). An XPS study on the chemical bond structure at the interface between SiO_x_N_y_ and N doped PET. J. Chem. Phys..

[20] Cho H.J., Kondo H., Ishikawa K., Sekine M., Hiramatsu M., Hori M. (2014). Density control of carbon nanowalls grown by CH_4_/H_2_ plasma and their electrical property. Carbon.

[21] Hannemann M., Hamann S., Burlacov I., Börner K., Spies H.J., Röpcke J. (2013). Langmuir probe and optical diagnostics of active screen N_2_-H_2_ plasma nitriding processes with admixture of CH_4_. Surf. Coat. Technol...

[22] Sankaran K.J., Kurian J., Chen H.C., Dong C.L., Lee C.Y., Tai N.H., Lin I.N. (2012). Origin of a needle-like granular structure for ultrananocrystalline diamond films grown in a N_2_/CH_4_ plasma. J. Phys. D Appl. Phys..

[23] Ndiaye A.A., Lago V. (2011). Optical spectroscopy investigation of N_2_-CH_4_ plasma jets simulating Titan atmospheric entry conditions, *Plasma Sources Sci*. Technol..

[24] Tahar R.B.H., Ban T., Ohya Y., Takahashi Y. (1998). Tin doped indium oxide thin films: Electrical property. J. Appl. Phys..

[25] Kim H., Gilmore C.M., Pique A., Horwitz J.S., Mattoussi H., Murata H., Kafafi Z.H., Chrisey D.B. (1999). Electrical, optical, and structural property of indium-tin-oxide thin films for organic light-emitting devices. J. Appl. Phys..

[26] Facchetti A., Marks T.J. (2010). Transparent Electronics: From Synthesis to Applications.

[27] Wang H. (2014). Preparation of High-Quality Indium Tin Oxide Film Used for Organic Light-Emitting Display. Ph.D. Thesis.

[28] Moulder J.F., Stickle W.F., Sobol P.E., Bomben K.D. (1995). Handbook of X-Ray Photoelectron Spectroscopy.

[29] Beamson G., Briggs D. (1992). High Resolution XPS of Organic Polymers: the Scienta ESCA3000 Database.

[30] Oyama S.T. (1996). The Chemistry of Transition Metal Carbides and Nitrides.

[31] Gusev A.I., Rempel A.A., Magerl A.J. (2001). Disorder and Order in Strongly Nonstoichiometric Compounds Transition Metal Carbides, Nitrides and Oxides.

[32] Wu Z., Wang B., Sun X. (2017). The Film Growth.

[33] Cao Z. (2011). Thin Film Growth: Physics, Materials Science and Applications.

[34] Lee H.C., Park O.O. (2004). Electron scattering mechanisms in indium-tin-oxide thin films: grain boundaryand ionized impurity scattering. Vacuum.

[35] Mei F., Yuan T., Li R., Qin K., Huang J. (2018). Microstructure evolution and grain orientation in ITO targets and their effects on the film characteristics. J. Mater. Sci. Mater. Electron..

[36] Sadofev S., Cho Y.J., Brandt O., Ramsteiner M., Calarco R., Riechert H., Erwin S.C., Galazka Z., Korytov M., Albrecht M. (2012). Growth of wurtzite InN on bulk In_2_O_3_(111) wafers. Appl. Phys. Lett..

[37] Hagleitner D.R., Menhart M., Jacobson P., Blomberg S., Schulte K., Lundgren E., Kubicek M., Fleig J., Kubel F., Puls C. (2012). Bulk and surface characterization of In_2_O_3_ (001) single crystals. Phys. Rev. B.

[38] Zhang K.H.L., Walsh A., Catlow C.R.A., Lazarov V.K., Egdell R.G. (2010). Surface energies control the self-organization of oriented In_2_O_3_ nanostructures on cubic zirconia. Nano Lett..

