# Supplementary material for: The Diffusion of Low-Energy Methyl Group on ITO Film Surface and Its Impact on Optical-Electrical Properties
Source: Materials (Basel). 2018 Oct 16;11(10):1991. doi: 10.3390/ma11101991 (PMC6213592; doi:10.3390/ma11101991)
Supplement: Supplementary file 1 [file materials-11-01991-s001.pdf]

Supplementary Materials for

The diffusion of Low-Energy Methyl Group on ITO Film Surface and its Impact on Optical-Electrical Properties

Shiping Zhao [1,†], Zhixuan Lv [1,†], Xuelin Guo [1], Chaoqian Liu [1], Hualin Wang [1,2], Weiwei Jiang [1], Shimin Liu [1], Nan Wang [1], Yunxian Cui [1], Wanyu Ding [1,2,*], Bing Han [2,*] and Dongying Ju [2,3]

[1] College of Materials Science and Engineering, Dalian Jiaotong University, Dalian 116028, China; zhaosp105@163.com (S.Z.); lzx_djtu@163.com (Z.L.); g1442944561@icloud.com (X.G.); cqliu@djtu.edu.cn (C.L.); whl@djtu.edu.cn (H.W.); jww@djtu.edu.cn (W.J.); lsm@djtu.edu.cn (S.L.); nwang@djtu.edu.cn (N.W.); dlcyx007@126.com (Y.C.)

[2] Liaoning Province Key Laboratory of Metallurgical Equipment and Process Control, University of Science and Technology Liaoning, Anshan 114051, China; dyju@sit.ac.jp

[3] Advance Science Center, Saitama Institute of Technology, Fukaya 369-0293, Japan

* Correspondence: dwysd@djtu.edu.cn (W.D.); hanbing@ustl.edu.cn (B.H.)

† These authors contributed equally to this work.

This supplementary file includes: Materials and Methods, Figure S1, Figure S2, Table S1, Supplementary Text, and Reference.

Materials and Methods

In our experiments, the quartz wafer was used as the substrate. For the cross-section crystal structure measurement by transmission electron microscopy (TEM), the polished Si (100) wafer was also used as the substrate. The quartz and Si (100) substrates were firstly ultrasonically pre-cleaned in the acetone, ethanol, and deionized water respectively, and then dried with N_2 gas. Finally the quartz and Si (100) substrates were loaded into the vacuum chamber, which was faced to the indium tin oxide (ITO) target. ITO target consisted of 10 wt.% SnO_2 and 90 wt.% In_2O_3 in composition, as well as 200×90 mm^2 in area. After the pressure in chamber was less than 9.9×10^{-4} Pa, the high purity Ar (99.99%) was introduced into the chamber as sputtering gas. The sputtering power was supplied by Pinnacle™ Plus+ 5 kW DC pulsed power supply unit. By controlling the sputtering power density on ITO target, ITO film was deposited onto the quartz and Si (100) substrates, simultaneously. The sputtering power density on ITO target was 1.67, 3.33, 5.00, and 6.66 W/cm^2 for ITO film (i), (ii), (iii), and (iv), respectively. The other deposition parameters were constant for all ITO films, just as shown in Table S1.

Table S1. The detailed parameters for ITO film deposition.

Parameter	Value
Working gas	Ar
Flow rate	20 sccm
Distance between target and substrate	12 cm
Pulse frequency	100 kHz
Reverse time	1 μs
Working pressure	0.6 Pa

During the experiment process, the surface morphology of ITO films before CH_x^+ group bombardment was measured by SPM9500-J3 atomic force microscopy (AFM) system, with 2.0 Hz scanning frequency in tapping mode. The collected data consisted of the height information on square 256×256 arrays of pixels.

Supplementary Text

About columnar crystal in ITO film with (100) preferred orientation

The cross-section TEM measurement was carried out onto ITO film (iv) deposited on Si (100) substrate, just as shown in Figure S1. From Figure S1, it can be seen clearly that ITO film (iv) consists of the columnar crystals, just as discussed in the manuscript. It should be noticed that ITO film was deposited on the quartz and Si (100) substrates, simultaneously. The cross-section TEM measurement was carried out onto ITO film deposited on Si (100) substrate, because the cross-section TEM sample of ITO film deposited on transparent quartz substrate couldn't be milled by Ar^+ ion beam. Based on our previous research work, in case of ITO films deposited on quartz and Si (100) substrates simultaneously, X-ray diffraction patterns of ITO films on above two kinds of substrate were almost same to each other. Then, ITO films on quartz and Si (100) substrates were with the same crystal structure and preferred orientation, just as shown in our previous reports [S1, S2]. Based on above discussion, it is believed that ITO film with (100) preferred orientation on quartz substrate also consists of the columnar crystals.

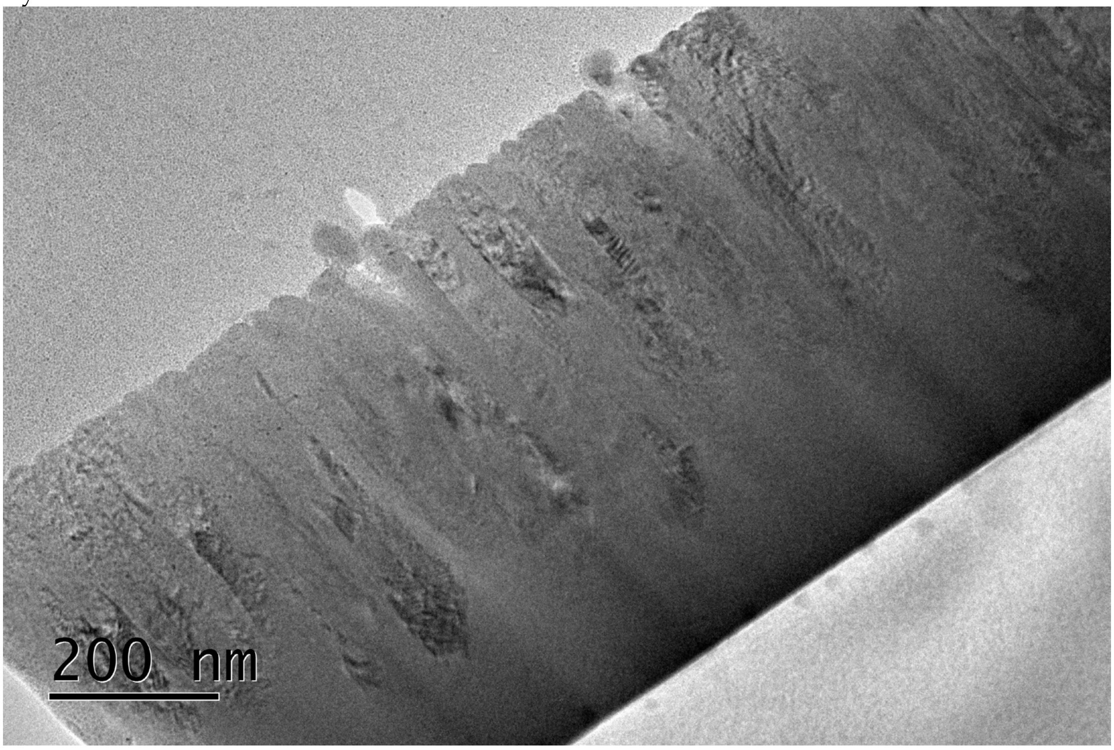

Figure S1. The cross-section TEM image of ITO film (iv) on Si (100) substrate.

About the thickness of the diffusion layer

In case of thickness of the diffusion layer, only 3D quantitative atom density distribution with nano-scale resolution ratio could provide the thickness result, directly. Actuall, 3D atom probe and/or time of flight secondary ion mass spectrometry (TOF-SIMS) techniques could provide 3D quantitative atom density distribution with nano-scale resolution [S3, S4]. In case of bulk materials, for 3D atom probe measurement and TOF-SIMS, it was easy to prepare filamentous specimen by focused ion beam system. But for ITO film on Si or quartz substrate, it was very difficult to prepare filamentous specimen for 3D atom probe measurement. So

until now, the authors haven't carried out 3D atom probe measurement for our ITO film. In case of TOF-SIMS measurement, frankly, the authors' affiliation has no ToF-SIMS system. The authors are finding help from the affiliations owning ToF-SIMS system. The authors will go on to design the further experiment and measurement to obtain the accurate thickness of diffusion layer.

About the surface morphology of ITO film

Figure S2 shows AFM images of ITO film (iv). From Figure S2 (a) and (b), it can be found that some island structures appear on the film surface. Then the root-mean-square (RMS) roughness was calculated by SPM9500-J3 AFM system, automatically. The results show that RMS roughness for (a)/(b) and (c)/(d) are 2.823 and 2.826 nm, respectively. Actually, RMS roughness keeps at constant after the scanning size larger than 1×1 μm^2 for all ITO films, which means the characteristic size is larger than 1×1 μm^2 for all ITO films. In order of ITO (i) to (iv), the value of RMS roughness gradually increased from 1.244 to 2.823 for scanning size of 1×1 μm^2 and from 1.386 to 2.826 for scanning size of 16×16 μm^2, respectively. The surface of ITO film (iv) is the roughest one, which is caused by the (100) preferred orientation and columnar crystals in film. But, it should be noticed that the surface morphology of all films was uniform and RMS roughness of all films was lower than 3 nm. Compared with the sputtering depth of 20-40 nm for XPS measurement, it was believed that the roughness of 3 nm could not influence the sputtering ratio during XPS measurement. Based on above discussion, it was believed that it took the similar time to remove the surface layer for all ITO films.

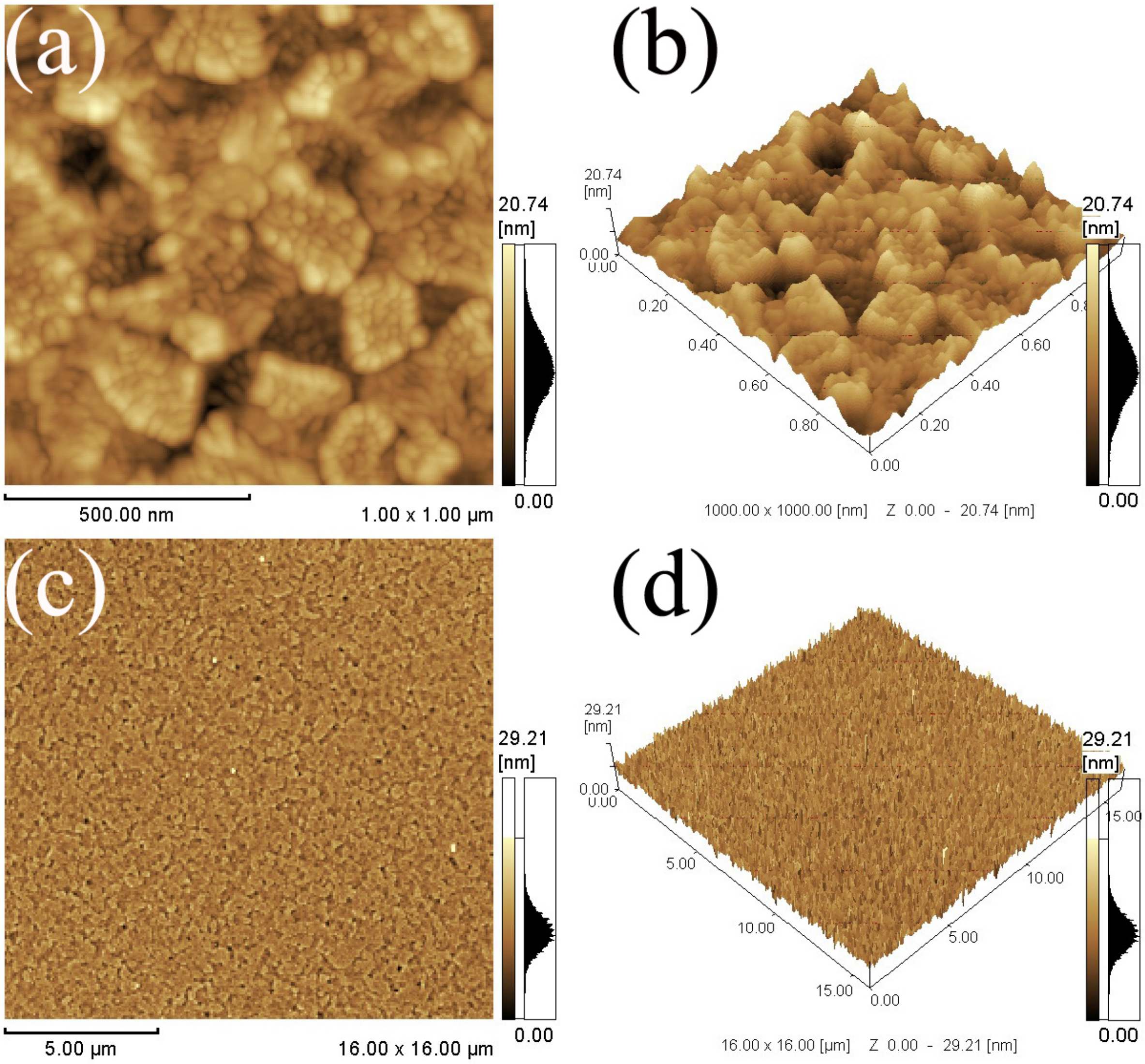

Figure S2. AFM images for ITO film (iv). (a) and (b) are the surface morphology with planar and 3D AFM images respectively, which the scanning size is 1×1 μm². (c) and (d) are the surface morphology with planar and 3D AFM images respectively, which the scanning size is 16×16 μm².

Reference

S1. Lv, Z.; Liu, J.; Wang, D.; Tao, H.; Chen, W.; Sun, H.; He, Y.; Zhang, X.; Qu, Z.; Han, Z.; Guo, X.; Zhao, S.; Cui, Y.; Wang, H.; Liu, S.; Liu, C.; Wang, N.; Jiang, W.; Chai, W.; Ding, W. A simple route to prepare (100) preferred orientation indium tin oxide film onto polyimide substrate by direct current pulsed magnetron sputtering, *Mater. Chem. Phys.* **2018**, *209*, 38.

S2. Chen, W.; Sun, H.; Jiang, W.; Wang, H.; Liu, S.; Liu, C.; Wang, N.; Cui, Y.; Chai, W.; Ding, W.; Han, B. A deep understanding of relationship between reverse time and growth of ITO film deposited by direct current pulsed sputtering, *Mater. Lett.* **2018**, *220*, 8.

S3. D. Blavette, A. Bostel, J. M. Sarrau, B. Deconihout, and A. Menand, An atom probe for three-dimensional tomography, Nature 363 (1993) 432-435.

S4. Benninghoven, A. Chemical-analysis of inorganic and organic-surfaces and thin-films by static time-of-flight secondary-ion mass-spectrometry (ToF-SIMS), *Angew. Chem. Int. Edit.* **1994**, *33*, 1023-1043.
